# The Cervical Lymph Node Positive Metastatic Probability Is a Significant Predictor of Survival for Oral Squamous Cell Carcinoma—A Nationwide Study

**DOI:** 10.3390/cancers17162704

**Published:** 2025-08-20

**Authors:** Li-Jen Liao, Cheng-Lin Lu, Yu-Ping Cheng, Ping-Chia Cheng, Yong-Chen Chen, Chun-Ju Chiang, Wen-Chung Lee, San-Lin You, Wan-Lun Hsu

**Affiliations:** 1 Department of Otolaryngology, Far Eastern Memorial Hospital, New Taipei City 220216, Taiwan; deniro@mail2000.com.tw (L.-J.L.); tgetptg@yahoo.com.tw (C.-L.L.); i.cruising@gmail.com (P.-C.C.); 2 Department of Electrical Engineering, Yuan Ze University, Taoyuan 320315, Taiwan; 3 Master Program of Big Data in Medical Health Care Industry, College of Medicine, Fu Jen Catholic University, New Taipei City 242062, Taiwan; fun10404@gmail.com (Y.-P.C.); 137159@mail.fju.edu.tw (Y.-C.C.); 4 Post-Baccalaureate Program in Nursing, Fu Jen Catholic University, New Taipei City 242062, Taiwan; 5 Graduate Institute of Epidemiology and Preventive Medicine, College of Public Health, National Taiwan University, Taipei 10055, Taiwan; ruru_chiang@cph.ntu.edu.tw (C.-J.C.); wenchung@ntu.edu.tw (W.-C.L.); 6 Genomics Research Center, Academia Sinica, Taipei 115201, Taiwan; 7 Department of Public Health, College of Medicine, Fu Jen Catholic University, New Taipei City 242062, Taiwan; 8 Data Science Center, College of Medicine, Fu Jen Catholic University, New Taipei City 242062, Taiwan

**Keywords:** oral cancer, histopathological features, survival, log odds of positive lymph nodes, lymph node density, national cancer registry

## Abstract

Background/Objectives: This study aimed to evaluate the prognostic significance of lymph node density (LND) and the log odds of positive lymph nodes (LODDS) in patients with oral squamous cell carcinoma (OSCC) using a nationwide database. Methods: A retrospective cohort study was conducted using the Taiwan Cancer Registry to identify patients diagnosed with OSCC who underwent surgery for both the primary tumor and neck dissection. Clinicopathological variables were collected, and survival outcomes were analyzed using Cox proportional hazards models. LND was categorized as negative, <0.05, and ≥0.05; LODDS was grouped into four categories: <−4, −4 to −3.5, −3.5 to −2.5, and ≥−2.5. Results: A total of 1643 female and 15,475 male patients were included, with a mean age of 57.4 years (range, 20–98 years). In multivariable Cox regression analyses, LND and LODDS were identified as independent prognostic factors for overall survival. Compared with patients with negative LND, the hazard ratios for LND < 0.05 and LND ≥0.05 were 2.12 (95% CI, 1.90–2.36) and 3.35 (95% CI, 3.05–3.67), respectively (*p* < 0.01). Similarly, relative to the lowest LODDS group (<−4), the hazard ratios for the higher categories were 1.51 (95% CI, 1.32–1.74) for −4 to −3.5, 2.30 (95% CI, 2.05–2.57) for −3.5 to −2.5, and 4.32 (95% CI, 3.85–4.86) for ≥−2.5 (*p* < 0.01). Conclusions: LND and LODDS are significant prognostic indicators in OSCC. Incorporating these lymph node–based metrics into prognostic models may enhance risk stratification and inform clinical decision-making.

## Simple Summary

Oral cancer is one of the most common cancers of the head and neck, and many patients are diagnosed at an advanced stage. Lymph node involvement is a major factor in predicting a patient’s outcome. This study used data from a large national cancer registry to examine two lymph node–based indicators: lymph node density and the log odds of positive lymph nodes. We found that both measures were associated with survival and helped identify high-risk patients among those who received surgical treatment. These findings suggest that lymph node–based assessment may support improved risk stratification in oral cancer management.

## 1. Introduction

Cancer is the leading cause of death worldwide [1]. Oral cancer, the most common malignancy of the head and neck region, is predominantly composed of squamous cell carcinomas (OSCCs) [2]. Despite aggressive treatment, the overall prognosis for OSCC remains poor, largely due to late-stage diagnosis at presentation [3].

Traditional prognostic factors include clinical stage [4], histological differentiation [5,6], tumor size, and treatment modality [3]. In addition, several research suggests that additional factors, such as perineural invasion [7,8,9], lymphatic or vascular invasion (LVI) [10], the number of involved regional lymph nodes (LNY) [11,12], distance to surgical margins [13,14], tumor depth [15,16,17], margin-to-depth ratio (MDR) [18,19], and extracapsular spread of lymph nodes, all of which may significantly impact patient survival.

Recently, cervical lymph node status has been recognized as a major prognostic factor for OSCC. Several studies have explored alternative nodal assessment methods, such as lymph node density (LND) [12] and the log odds of positive lymph nodes (LODDS) [20,21], suggesting their potential prognostic value. Chang et al. reported that an LND cutoff of 0.05 is associated with survival outcomes, with 5-year OS rates of 48.4% for LND < 0.05 and 30.4% for LND ≥ 0.05, whereas the 5-year disease-free survival (DFS) rates were 42.7% and 17.3%, respectively [22]. Lee et al. analyzed 347 OSCC patients and reported that those in the highest LODDS group had significantly lower 5-year disease-specific survival than did those in the lowest LODDS group (adjusted HR 5.42, 95% CI 3.19–9.12) [23].

Since many previous studies were limited to data from individual hospitals or focused on only a few pathological factors, this study leveraged the Taiwan National Cancer Database to analyze a wide range of clinical and pathological variables comprehensively. By utilizing this large-scale, population-based dataset, this study aimed to provide a more robust evaluation of the prognostic significance of both LND and LODDS in patients with OSCC, offering valuable insights into their respective roles in survival prediction.

## 2. Materials and Methods

### 2.1. Study Design and Data Source

This retrospective cohort study utilized data from the Taiwan Cancer Registry (TCR) long-form database. The TCR is a nationwide population-based registry that collects detailed information on cancer diagnosis, staging, and treatment. Follow-up was conducted through linkage with national death records until 31 December 2023.

### 2.2. Study Population

**Figure 1:**
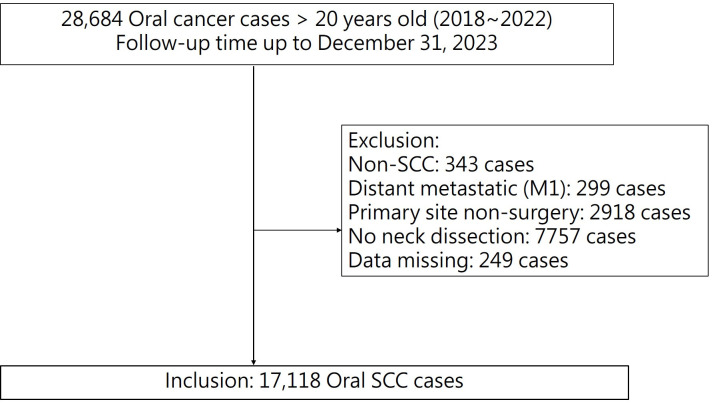
Flowchart of the patient enrollment and selection process.

Adult patients diagnosed with OSCC between 2018 and 2022 were identified from the Taiwan Cancer Registry using topography codes from the *International Classification of Diseases for Oncology*, Third Edition (ICD-O-3), including C00 (lip), C02 (tongue), C03 (gingiva), C04 (floor of mouth), C05 (palate), and C06 (other and unspecified parts of mouth). Cases with codes C02.4 (lingual tonsil), C05.1 (soft palate), and C05.2 (uvula) were excluded to avoid potential misclassification of oropharyngeal cancers. Pathological staging was based on the eighth edition of the American Joint Committee on Cancer (AJCC) staging system. Eligible patients were those who received surgical treatment for both the primary tumor and cervical lymph nodes. Patients were excluded if they presented with distant metastases at initial diagnosis, had multiple primary malignancies, lacked surgical intervention, or had incomplete pathological or staging information. A detailed flowchart of the case selection process is presented in Figure 1. This study was approved by the Institutional Review Board (IRB No: C110196), and informed consent was waived due to the use of de-identified registry data.

### 2.3. Study Variables

Demographic, clinical, and pathological variables were collected, including age, sex, body mass index (BMI), Eastern Cooperative Oncology Group (ECOG) performance status, tumor subsite, AJCC stage, histological grade, perineural invasion (PNI), lymphovascular invasion (LVI), depth of invasion (DOI), extracapsular spread (ECS), surgical margin status, and treatment modality. A small proportion of clinical and pathological variables contained missing values due to incomplete documentation in the registry. These missing data were not imputed and were excluded from analyses involving the corresponding variables. BMI was classified according to the Ministry of Health and Welfare in Taiwan as underweight (<18.5 kg/m^2^), normal (18.5–23.9), overweight (24.0–26.9), and obese (≥27.0) [24]. ECOG performance status was defined on a scale from 0 (fully active) to 4 (completely disabled) [25]. Two lymph node-positive probability indicators, namely, LND and LODDS, were also utilized. LND was calculated as the ratio of positive to total dissected lymph nodes and categorized as negative (0), <0.05, and ≥0.05. These cut-off values were determined based on thresholds used in previous study [22]. The LODDS was defined as log{(number of positive nodes + 0.5)/(number of negative nodes + 0.5)} [20]. LODDS were divided into four categories: <−4, −4 to −3.5, −3.5 to −2.5, and >−2.5, based on approximate quartiles rounded for interpretability.

Treatment characteristics included surgical margin status and lymph node yield (LNY), defined as the total number of cervical lymph nodes removed. Treatment modality was categorized into four groups: surgery alone, surgery plus adjuvant radiotherapy (RT), surgery plus adjuvant chemotherapy (CT), and surgery plus adjuvant chemoradiotherapy (CRT).

### 2.4. Statistical Analysis

Categorical variables were expressed as counts and percentages, and continuous variables were presented as the means (±standard deviations; SDs). Follow-up time was defined as the period from the date of diagnosis to the date of death or 31 December 2023, whichever occurred first. Five-year survival rates were estimated using the Kaplan–Meier method, and differences between groups were assessed using the log-rank test. Cox proportional hazards regression models were applied to evaluate the independent effects of clinical and pathological variables on overall survival (OS) and disease-specific survival (DSS), with results presented as hazard ratios (HRs) and 95% confidence intervals (CIs). Trend tests were conducted to assess dose-response relationships across ordered categories. All the statistical analyses were carried out using STATA13 (Stata Corporation, College Station, TX, USA).

## 3. Results

There were 28,685 patients diagnosed with oral cancer between 2018 and 2022. After excluding patients who did not undergo primary tumor resection, lacked neck dissection, had distant metastasis at diagnosis, or had incomplete clinical or pathological data, 17,118 patients were included in the analysis, as shown in Figure 1. Table 1 summarizes the baseline characteristics of the included patients, including survival outcomes. By the end of follow-up on 31 December 2023, 4708 patients had died. The five-year survival rate was 66.51% (95% CI: 64.59–66.40). The cohort had a mean age of 57.4 years (range: 20–98 years), and 90.4% of patients were male. In terms of BMI distribution, 27.7% were overweight, 30.3% were obese, and 4.2% were underweight.

The most common tumor subsites were the tongue (36.2%) and cheek mucosa (32.0%). Histologically, 65.2% of the tumors were moderately differentiated, with a mean tumor size of 30.6 mm with a standard deviation of 17.4 mm. Positive PNI and LVI were observed in 29.3% and 18.2% of the patients, respectively. An LND ≥ 0.05 was observed in 11.2% of patients and was associated with a significantly lower five-year survival rate (37.5%) compared to those with LND < 0.05 (54.7%) and LND = 0 (75.3%). Similarly, increasing LODDS values were associated with poorer five-year survival rates, decreasing from 78.0% in LODDS < −4 to 40.4% in LODDS ≥ −2.5.

The surgical margins were positive in 5.6% of the patients. The LNY exceeded 30 in 42.2% of patients, whereas 19.2% had fewer than 15 nodes harvested. Treatment modalities included surgery alone (43.0%), surgery plus RT (14.0%), surgery plus CT (6.7%), and surgery plus CRT (36.3%). Five-year survival rates varied according to clinical factors, with poorer prognoses observed in older age groups, patients with advanced tumor stages, and those with adverse pathological features such as elevated LND and higher LODDS and positive margins.

**Figure 2:**
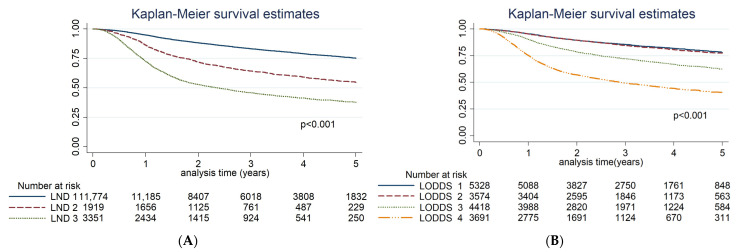
Kaplan–Meier curves for overall survival stratified by (**A**) lymph node density (LND) and (**B**) log odds of positive lymph nodes (LODDS). LND was divided into three groups: 0, <0.05, and ≥0.05. LODDS was categorized into four groups: <−4, −4 to −3.5, −3.5 to −2.5, and >−2.5.

Table 2 presents the results of univariate and multivariate Cox regression analyses for OS. Univariate analysis revealed significant associations between OS and factors such as age (*p* < 0.01), BMI (*p* < 0.01), and ECOG performance status (*p* < 0.01). Among tumor subsites, lip cancer has the most favorable prognosis. Other significant factors influencing OS included the AJCC stage, histological grade, PNI, LVI, LND, and LODDS. Treatment factors such as surgical margin status, LNY, and intensive treatment were also significant predictors of OS. The Kaplan–Meier survival curves (Figure 2) revealed significant differences in OS based on varying levels of LND and LODDS (log-rank test, *p* < 0.01), which is reflected in the univariate results in Table 2. In the multivariable analysis, Model 1 included LND and showed that, compared with patients with LND = 0, the hazard ratios (HRs) for LND < 0.05 and LND ≥ 0.05 were 2.12 (95% CI: 1.90–2.36) and 3.35 (95% CI: 3.05–3.67), respectively (*p* < 0.01). Model 2 included LODDS and demonstrated that, compared with patients with LODDS < −4, the HRs for LODDS −4 to −3.5, −3.5 to −2.5, and ≥−2.5 were 1.51 (95% CI: 1.32–1.74), 2.30 (95% CI: 2.05–2.57), and 4.32 (95% CI: 3.85–4.86), respectively (*p* < 0.01). To further evaluate whether LND and LODDS serve as independent prognostic indicators, we conducted a subgroup analysis restricted to patients with pathologically confirmed node-positive (pN+) disease (Supplemental Table S1). In this cohort, both LND and LODDS remained significant predictors of overall survival. Patients with LND ≥ 0.05 had an adjusted HR of 1.74 (95% CI: 1.56–1.93), and those with LODDS > −2.5 had an adjusted HR of 1.96 (95% CI: 1.26–3.05), compared to their respective reference categories.

Table 3 presents the results of the Cox proportional hazards regression analysis for DSS. Consistent with previous models, both LND and LODDS remained significant prognostic factors for DSS. Compared with the reference group, patients with LND ≤ 0.05 had a significantly increased risk of disease-specific mortality (adjusted HR = 2.31, 95% CI: 2.04–2.61, *p* < 0.001). The risk was even greater among those with LND > 0.05 (adjusted HR = 3.81, 95% CI: 3.43–4.23, *p* < 0.001). With respect to the LODDS, a clear gradient of increasing hazard was observed. Using LODDS < −4 as the reference group, patients with LODDS −4 to −3.5 had a moderately elevated risk of DSS (adjusted HR = 1.57, 95% CI: 1.33–1.85, *p* < 0.001), which further increased in those with LODDS −3.5 to −2.5 (adjusted HR = 2.50, 95% CI: 2.20–2.84, *p* < 0.001) and LODDS ≥ −2.5 (adjusted HR = 4.86, 95% CI: 4.25–5.56, *p* < 0.001).

## 4. Discussion

In this study, LND and LODDS were identified as significant and independent prognostic indicators for both OS and DSS. These findings remained robust even after adjusting for patient-, tumor-, and treatment-related factors in multivariable Cox regression models. The significant dose–response relationships observed, as evidenced by the trend tests, further reinforce the clinical relevance of these lymph node-based metrics in risk stratification and prognosis assessment.

In terms of patient factors, age, BMI, and ECOG performance status were independently associated with the prognosis of patients with OSCC, which is consistent with findings from previous studies [26]. Compared with tongue cancer, gum and palate cancers are associated with significantly poorer overall survival (Table 2). Multiple-variable analysis has revealed that palate cancer has a significantly poorer overall survival than tongue cancer does. These results suggest that other clinicopathological variables may influence survival outcomes. Further analysis of margin status revealed that tumors located in the hard palate had a notably higher positive margin rate (18.7%) than other subsites did (all <10%), indicating potential challenges in surgical resection and the need for further investigation of subsite-specific tumor behavior.

In LNY analysis, the cutoff value has shown considerable variation in previous studies [11,12]. In our study, we observed that an LNY greater than 15 was associated with a better prognosis. However, LNY values exceeding 30 did not yield further survival benefits in multivariate Cox regression analysis. This observation aligns with the findings of Lee et al., who reported a similar trend [27]. Moreover, patients who underwent more extensive neck dissection (LNY > 40) did not have an improved prognosis.

LND and LODDS have both been shown to be significant prognostic factors for OS in OSCC patients. LODDS, which adds 0.5 to both the number of positive lymph nodes and total neck lymph nodes, offers an advantage by distinguishing between patients without positive lymph nodes, even when total LNY is low [28]. Previous studies have been limited by smaller sample sizes, but in our study, which utilized a large database, we classified the LODDS into four levels. Consistent with prior research, we found that LODDS remained an independent prognostic factor for overall survival. Compared with patients with LODDS < −4, the hazard ratios for LODDS −4 to −3.5, −3.5 to −2.5, and >−2.5 were 1.51 (95% CI: 1.32–1.74), 2.30 (95% CI: 2.05–2.57), and 4.32 (95% CI: 3.85–4.86), respectively (Table 2). A significant dose–response relationship was observed across LODDS categories in the trend test. To assess the discrimination power of the multivariate regression models, we employed the likelihood ratio chi-square (LR x^2^) statistic. A higher LR x^2^ value indicates a better model fit and predictive accuracy [29]. Our model comparison for LODDS and LND revealed that LODDS had a higher LR x^2^ than LND for OS, suggesting that LODDS may offer slightly better predictive performance. However, the results for both indicators were quite similar, which aligns with findings from a previous study [30]. To further validate these findings, we conducted a subgroup analysis limited to node-positive patients. Both LND and LODDS remained significant predictors of overall survival in this cohort, reinforcing their independent prognostic value even among patients with confirmed lymph node metastases (see Supplemental Table S1). In our opinion, both LND and LODDS serve as critical prognostic indicators for OS and DSS. LND, being simpler and more practical to measure, is readily applicable in clinical settings. In contrast, the LODDS, which involves a log-transformation of the lymph node data, provides a more refined prognostic tool, although it is more complex to calculate. Ultimately, we suggest that the concept of lymph node-based metrics, particularly LND and LODDS, should be more widely integrated into future clinical care for OSCC patients, as they have demonstrated strong prognostic value.

There are several limitations to this study that should be considered. First, our analysis was limited to patients who underwent primary surgery with neck dissection, which may not be fully representative of all OSCC patients. Second, there was variability in the types of neck lymph node dissection and surgical techniques used across the study population, which could introduce bias or affect the generalizability of our findings. Third, various studies have employed different cutoff values and classification schemes for LODDS, which may limit the comparability of our results to those of other studies. Fourth, some clinical and pathological variables contained missing values due to incomplete documentation in the registry. Records with missing values were excluded from analyses involving the corresponding variables. Although the proportion of missing data was relatively small, its potential impact on the results cannot be entirely ruled out. Lastly, DFS was not included in our analysis, as recurrence data in the Taiwan Cancer Registry were not yet sufficiently complete and consistent during the study period. Therefore, in future studies, these factors should be considered to better refine and standardize prognostic assessments for OSCC.

## 5. Conclusions

This study utilized a large database to validate the associations of various factors—patient, tumor, and treatment characteristics—with survival outcomes in patients with OSCC. Our findings confirm that lymph node positivity indicators, specifically LND and LODDS, are significant and independent predictors of both OS and DSS in patients with OSCC. These results underscore the prognostic value of lymph node-based metrics in the clinical management of OSCC patients.

## Figures and Tables

**Table 1:** Characteristics of the recruited patients (n = 17,118).

Variables	Numbers (%)	Death Numbers	5-Year Survival Rate (95% CI)
Patient characteristics			
Age (year)			
<40	793 (4.6)	166	73.6 (69.5–77.2)
40–49	3335 (19.5)	854	68.8 (66.8–70.6)
50–59	5762 (33.7)	1414	69.3 (67.8–70.8)
60–69	5050 (29.5)	1404	64.4 (62.6–66.1)
≥70	2178 (12.7)	870	49.4 (46.5–52.2)
Mean ± SD	57.36 ± 10.84		
Gender			
Female	1643 (9.6)	424	68.6 (65.7–71.3)
Male	15,475 (90.4)	4284	65.2 (64.2–66.2)
BMI ^a^			
<18.5	714 (4.2)	1976	60.6 (59.1–62.1)
18.5–23.9	6204 (36.2)	308	48.9 (44.3–53.3)
24–26.9	4736 (27.7)	1218	67.7 (66.0–69.4)
≥27	5182 (30.3)	1125	71.9 (70.2–73.4)
Mean ± SD	25.25 ± 4.42		
ECOG PS ^b^			
0	9389 (62.7)	2306	69.4 (68.2–70.6)
1	5088 (29.7)	1579	59.8 (58.0–61.6)
2	349 (2.0)	182	40.7 (34.5–46.8)
3/4	147 (0.9)	73	44.7 (35.3–53.7)
Tumor characteristics			
Subsite			
Tongue	6191 (36.2)	1672	66.3 (64.8–67.8)
Cheek Mucosa	5478 (32.0)	1392	68.2 (66.7–69.8)
Gum	3031 (17.7)	947	60.5 (58.2–62.7)
Lip	745 (4.4)	167	69.8 (65.1–74.0)
Retro-molar area	683 (4.0)	190	67.2 (62.7–71.3)
Floor of mouth	692 (4.0)	214	60.8 (55.9–65.3)
Palate	298 (1.7)	126	46.6 (38.9–53.8)
p-T classification (AJCC8) ^c^			
I	3812 (22.3)	427	83.4 (81.7–85.0)
II	4872 (28.5)	1042	72.2 (70.5–73.7)
III	2845 (16.6)	934	60.2 (57.9–62.3)
IV	5589 (32.7)	2305	50.2 (48.5–51.9)
Tumor size (mm)			
Mean ± SD	30.6 ±17.4		
Histological grade ^d^			
Well differentiated	4446 (26.0)	844	74.6 (72.9–76.2)
Moderately differentiated	11,152 (65.2)	3227	64.0 (62.8–65.1)
Poorly differentiated	1264 (7.4)	578	46.4 (42.9–49.8)
PNI ^e^			
Negative	11,193 (65.4)	2303	72.6 (71.5–73.7)
Positive	5014 (29.3)	2166	49.7 (48.0–51.4)
LVI ^f^			
Negative	13,041 (76.2)	2975	70.5 (69.5–71.5)
Positive	3113 (18.2)	1470	45.4 (43.3–47.5)
LND ^g^			
0	11,836 (69.1)	2160	75.3 (74.3–76.3)
<0.05	3351 (19.6)	709	54.7 (51.8–57.4)
≥0.05	1919 (11.2)	1815	37.5 (35.5–39.6)
LODDS ^h^			
<−4	5328 (31.1)	865	78.0 (76.5–79.5)
−4 to −3.5	3574 (20.9)	599	77.3 (75.4–79.0)
−3.5 to −2.5	4418 (25.8)	1308	62.5 (60.6–64.3)
>−2.5	3691 (21.6)	1890	40.4 (0.39–0.42)
Mean ± SD	−3.3 ± 1.1		
Treatment characteristics			
Surgical margin (mm) ^i^			
positive	962 (5.6)	522	37.2 (33.3–41.1)
0.1–0.9	172 (1.0)	67	55.1 (46.2–63.1)
1–1.9	2118 (12.4)	765	55.3 (52.5–58.0)
2–2.9	1732 (10.1)	496	63.2 (60.1–66.0)
3–3.9	1827 (10.7)	489	66.8 (64.0–69.5)
4–4.9	1587 (9.3)	361	69.9 (66.8–72.8)
≥5	7227 (42.2)	1630	71.1 (69.7–72.4)
Mean ± SD	4.5 ± 3.5		
LNY ^j^			
1–14	3280 (19.2)	1047	59.9 (57.7–62.0)
15–29	6542 (38.2)	1558	69.6 (68.2–71.0)
≥30	7226 (42.2)	2082	64.5 (63.0–65.8)
Mean ± SD	30.8 ± 18.5		
Treatment			
Surgery	7356 (43.0)	1340	76.4 (75.2–77.7)
Surgery + RT	2400 (14.0)	649	51.7 (47.9–55.3)
Surgery + CT	1146 (6.7)	465	54.8 (53.2–56.4)
Surgery + CRT	6216 (36.3)	2254	65.6 (63.2–68.0)

Abbreviations: BMI: body mass index; PNI: perineural invasion; LND: lymph node density; LNY: lymph node yield; DOI: depth of invasion; ENE: extracapsular spread; ECOG PS: ECOG Performance Status Scale; RT: radiotherapy; CT: chemotherapy; CRT: chemoradiotherapy; NA: not available. ^a^ Data for the BMI were missing for 282 subjects. ^b^ Data for the ECOG PS were missing for 2145 subjects. ^c^ Data for the p-T classification (AJCC8) were missing for 250 subjects. ^d^ Data for the histological grade were missing for 256 subjects. ^e^ Data for the PNI were missing for 911 subjects. ^f^ Data for the LVI were missing for 964 subjects. ^g^ Data for the LND were missing for 12 subjects. ^h^ Data for the LODDS were missing for 107 subjects. ^i^ Data for the surgical margin were missing for 1493 subjects. ^j^ Data for the surgical margin were missing for 70 subjects.

**Table 2:** Univariate and multivariate Cox regression analyses for overall survival.

	Univariate Model		Multivariable Model 1		Multivariable Model 2	
Variables	HR (95% CI)	*p* Value	HR (95% CI)	*p* Value	HR (95% CI)	*p* Value
Patient characteristics						
Age (year)						
<40	1.00		1.00		1.00	
40–50	1.28 (1.08–1.51)	0.004	1.25 (1.02–1.52)	0.031	1.28 (1.05–1.56)	0.014
50–60	1.24 (1.06–1.46)	0.008	1.28 (1.05–1.55)	0.013	1.32 (1.09–1.59)	0.005
60–70	1.47 (1.25–1.72)	<0.001	1.48 (1.22–1.79)	<0.001	1.55 (1.28–1.89)	<0.001
≥70	2.37 (2.00–2.79)	<0.001	2.29 (1.87–2.80)	<0.001	2.34 (1.91–2.86)	<0.001
test for trend *p*	<0.001		<0.001		<0.001	
Gender						
Female	1.00		1.00		1.00	
Male	1.05 (0.95–1.16)	0.372	1.15 (1.03–1.30)	0.017	1.17 (1.04–1.32)	0.008
BMI						
18.5–23.9	1.00		1.00		1.00	
<18.5	1.50 (1.33–1.69)	<0.001	1.35 (1.17–1.55)	<0.001	1.42 (1.23–1.63)	<0.001
24–26.9	0.77 (0.72–0.83)	<0.001	0.86 (0.79–0.93)	<0.001	0.87 (0.80–0.95)	0.002
≥27	0.63 (0.59–0.68)	<0.001	0.84 (0.77–0.91)	<0.001	0.83 (0.76–0.91)	<0.001
test for trend *p*	<0.001		<0.001		<0.001	
ECOG PS						
0	1.00		1.00		1.00	
1	1.37 (1.28–1.46)	<0.001	1.17 (1.09–1.26)	<0.001	1.17 (1.09–1.26)	<0.001
2	2.57 (2.21–2.98)	<0.001	1.65 (1.40–1.96)	<0.001	1.60 (1.35–1.89)	<0.001
3/4	2.47 (1.96–3.12)	<0.001	1.73 (1.34–2.24)	<0.001	1.62 (1.26–2.10)	<0.001
test for trend *p*	<0.001		<0.001		<0.001	
Tumor characteristics						
Sub-site						
Tongue	1.00		1.00		1.00	
Lip	0.78 (0.67–0.92)	0.002	1.02 (0.84–1.23)	0.853	1.06 (0.88–1.28)	0.515
Cheek Mucosa	0.92 (0.86–0.99)	0.020	0.97 (0.89–1.06)	0.527	0.97 (0.89–1.06)	0.531
Retro-molar area	1.02 (0.88–1.18)	0.808	0.95 (0.79–1.13)	0.537	0.99 (0.83–1.18)	0.891
Floor of mouth	1.14 (0.99–1.31)	0.072	1.08 (0.92–1.28)	0.345	1.07 (0.91–1.26)	0.431
Gum	1.19 (1.10–1.29)	<0.001	0.90 (0.81–1.00)	0.042	0.93 (0.84–1.03)	0.178
Palate	1.65 (1.38–1.98)	<0.001	1.25 (1.00–1.57)	0.049	1.30 (1.04–1.63)	0.024
p-T (AJCC8)						
I	1.00		1.00		1.00	
II	2.01 (1.80–2.25)	<0.001	1.65 (1.44–1.90)	<0.001	1.58 (1.37–1.81)	<0.001
III	3.36 (3.00–3.76)	<0.001	2.63 (2.26–3.06)	<0.001	2.48 (2.13–2.88)	<0.001
IV	4.72 (4.26–5.23)	<0.001	3.78 (3.26–4.38)	<0.001	3.66 (3.16–4.23)	<0.001
test for trend *p*	<0.001		<0.001		<0.001	
Histological grade						
Well differentiated	1.00		1.00		1.00	
Moderately differentiated	1.64 (1.52–1.77)	<0.001	1.18 (1.08–1.30)	<0.001	1.19 (1.08–1.30)	<0.001
Poorly differentiated	3.07 (2.77–3.42)	<0.001	1.70 (1.49–1.93)	0.001	1.68 (1.48–1.91)	<0.001
test for trend *p*	<0.001		<0.001		<0.001	
PNI						
Negative	1.00		1.00		1.00	
Positive	2.55 (2.40–2.70)	<0.001	1.46 (1.35–1.57)	<0.001	1.47 (1.36–1.59)	<0.001
LVI						
Negative	1.00		1.00		1.00	
Positive	2.58 (2.42–2.74)	<0.001	1.15 (1.06–1.24)	0.001	1.11 (1.02–1.20)	0.014
LND						
0	1.00		1.00		1.00	
≤0.05	2.31 (2.12–2.51)	<0.001	2.12 (1.90–2.36)	<0.001		
>0.05	4.21 (3.95–4.48)	<0.001	3.35 (3.05–3.67)	<0.001		
LODDS						
<−4	1.00		1.00		1.00	
−4 to −3.5	1.03 (0.93–1.15)	0.548			1.51 (1.32–1.74)	<0.001
−3.5 to −2.5	1.99 (1.82–2.17)	<0.001			2.30 (2.05–2.57)	<0.001
>−2.5	4.31 (3.98–4.67)	<0.001			4.32 (3.85–4.86)	<0.001
test for trend *p*	<0.001				<0.001	
Treatment characteristics						
Surgical margin (mm)						
positive	1.00		1.00		1.00	
0.1–0.9	0.63 (0.49–0.81)	<0.001	0.94 (0.71–1.26)	0.681	0.93 (0.70–1.24)	0.632
1–1.9	0.56 (0.50–0.63)	<0.001	0.81 (0.71–0.92)	0.001	0.78 (0.69–0.89)	<0.001
2–2.9	0.42 (0.37–0.47)	<0.001	0.73 (0.63–0.84)	<0.001	0.71 (0.62–0.82)	<0.001
3–3.9	0.38 (0.34–0.43)	<0.001	0.68 (0.59–0.79)	<0.001	0.67 (0.58–0.77)	<0.001
4–4.9	0.32 (0.28–0.37)	<0.001	0.65 (0.55–0.75)	<0.001	0.64 (0.55–0.75)	<0.001
≥5	0.31 (0.29–0.35)	<0.001	0.61 (0.54–0.69)	<0.001	0.60 (0.53–0.67)	<0.001
test for trend *p*	<0.001		<0.001		<0.001	
LNY						
1–14	1.00		1.00		1.00	
15–29	0.71 (0.66–0.77)	<0.001	1.08 (0.98–1.20)	0.132	0.74 (0.67–0.81)	<0.001
≥30	0.91 (0.84–0.98)	0.013	1.67 (1.51–1.85)	<0.001	0.86 (0.78–0.95)	0.002
test for trend *p*	0.703		<0.001		<0.001	
Treatment						
Surgery	1.00		1.00		1.00	
Surgery + RT	1.53 (1.39–1.68)	<0.001	0.74 (0.66–0.83)	<0.001	0.67 (0.60–0.75)	<0.001
Surgery + CT	2.81 (2.53–3.12)	<0.001	1.22 (1.07–1.40)	0.003	1.16 (1.02–1.33)	0.029
Surgery + CRT	2.30 (2.15–2.47)	<0.001	0.59 (0.53–0.65)	<0.001	0.53 (0.48–0.59)	<0.001

Abbreviations: PNI: perineural invasion; LVI: lymphovascular invasion; LND: lymph node density; RT: radiotherapy; CT: chemotherapy; CRT: chemoradiotherapy; ECOG Performance Status Scale; NA: not available.

**Table 3:** Univariate and multivariate Cox regression for disease-specific survival.

	Univariate Model		Multivariable Model 1		Multivariable Model 2	
Variables	HR (95% CI)	*p* Value	HR (95% CI)	*p* Value	HR (95% CI)	*p* Value
Patient characteristics						
Age(year)						
<40	1		1		1	
40–50	1.17 (0.98–1.40)	0.076	1.14 (0.92–1.40)	0.236	1.17 (0.95–1.44)	0.136
50–60	1.08 (0.91–1.29)	0.357	1.10 (0.90–1.35)	0.355	1.14 (0.93–1.39)	0.216
60–70	1.20 (1.01–1.42)	0.042	1.18 (0.96–1.45)	0.109	1.25 (1.02–1.53)	0.033
≥70	1.81 (1.52–2.17)	<0.001	1.76 (1.41–2.18)	<0.001	1.79 (1.44–2.22)	<0.001
test for trend *p*	<0.001		<0.001		<0.001	
Gender						
Female	1		1		1	
Male	1.02 (0.91–1.14)	0.705	1.07 (0.94–1.23)	0.294	1.09 (0.95–1.24)	0.203
BMI						
18.5–23.9	1		1		1	
<18.5	1.53 (1.26–1.66)	<0.001	1.30 (1.11–1.52)	0.001	1.37 (1.17–1.61)	<0.001
24–26.9	0.81 (0.78–0.92)	<0.001	0.91 (0.83–1.00)	0.048	0.93 (0.85–1.02)	0.130
≥27	0.64 (0.63–0.75)	<0.001	0.84 (0.76–0.93)	0.001	0.84 (0.76–0.93)	<0.001
test for trend *p*	<0.001		<0.001		<0.001	
ECOG PS						
0	1		1		1	
1	1.33 (1.23–1.43)	<0.001	1.13 (1.04–1.23)	0.003	1.13 (1.05–1.23)	0.002
2	2.51 (2.11–2.99)	<0.001	1.65 (1.36–2.01)	<0.001	1.58 (1.30–1.92)	<0.001
3/4	2.41 (1.84–3.15)	<0.001	1.63 (1.21−2.20)	0.001	1.49 (1.10–2.01)	0.010
test for trend *p*	<0.001		<0.001		<0.001	
Tumor characteristics						
Sub-site						
Tongue	1		1		1	
Lip	0.73 (0.60–0.88)	0.001	1.03 (0.82–1.29)	0.819	1.08 (0.86–1.35)	0.506
Cheek Mucosa	0.94 (0.87–1.02)	0.124	1.01 (0.91–1.11)	0.897	1.01 (0.91–1.11)	0.897
Retro-molar area	0.90 (0.75–1.08)	0.258	0.87 (0.70–1.08)	0.197	0.91 (0.74–1.12)	0.383
Floor of mouth	1.01 (0.85–1.20)	0.871	0.99 (0.81–1.20)	0.902	0.97 (0.79–1.18)	0.739
Gum	1.19 (1.09–1.30)	<0.001	0.89 (0.79–1.01)	0.062	0.93 (0.83–1.05)	0.232
Palate	1.55 (1.25–1.91)	<0.001	1.22 (0.94–1.58)	0.141	1.27 (0.97–1.64)	0.077
p-T (AJCC8)						
I	1		1		1	
II	2.44 (2.11–2.81)	<0.001	1.93 (1.61–2.31)	<0.001	1.83 (1.53–2.19)	<0.001
III	4.47 (3.87–5.16)	<0.001	3.19 (2.64–3.85)	<0.001	2.97 (2.46–3.59)	<0.001
IV	6.48 (5.68–7.40)	<0.001	4.73 (3.93–5.69)	<0.001	4.54 (3.78–5.46)	<0.001
test for trend *p*	<0.001		<0.001		<0.001	
Histological grade						
Well differentiated	1		1		1	
Moderately differentiated	1.82 (1.67–2.00)	<0.001	1.23 (1.10–1.37)	<0.001	1.24 (1.11–1.38)	<0.001
Poorly differentiated	3.61 (3.20–4.08)	<0.001	1.79 (1.54–2.07)	<0.001	1.76 (1.52–2.05)	<0.001
test for trend *p*	<0.001		<0.001		<0.001	
PNI						
Negative	1		1		1	
Positive	2.93 (2.74–3.13)	<0.001	1.53 (1.40–1.67)	<0.001	1.54 (1.41–1.68)	<0.001
LVI						
Negative	1		1		1	
Positive	2.83 (2.63–3.03)	<0.001	1.15 (1.05–1.26)	0.002	1.11 (1.01–1.22)	0.027
LND						
0	1		1			
≤0.05	2.70 (2.45–2.97)	<0.001	2.31 (2.04–2.61)	<0.001		
>0.05	5.07 (4.71–5.44)	<0.001	3.81 (3.43–4.23)	<0.001		
LODDS						
<−4	1				1	
−4 to −3.5	1.01 (0.89–1.14)	0.886			1.57 (1.33–1.85)	<0.001
−3.5 to −2.5	2.14 (1.94–2.37)	<0.001			2.50 (2.20–2.84)	<0.001
>−2.5	4.88 (4.11–5.36)	<0.001			4.86 (4.25–5.56)	<0.001
test for trend *p*	<0.001				<0.001	
Treatment characteristics						
Surgical margin						
positive	1		1		1	
0.1–0.9 mm	0.60 (0.45–0.80)	0.002	0.97 (0.71–1.32)	0.832	0.95 (0.70–1.31)	0.773
1–1.9 mm	0.54 (0.48–0.62)	<0.001	0.80 (0.70–0.92)	0.002	0.78 (0.67–0.89)	0.014
2–2.9 mm	0.37 (0.32–0.43)	<0.001	0.69 (0.58–0.80)	<0.001	0.67 (0.57–0.79)	0.002
3–3.9 mm	0.36 (0.31–0.41)	<0.001	0.69 (0.59–0.81)	<0.001	0.68 (0.58–0.80)	0.003
4–4.9 mm	0.29 (0.25–0.34)	<0.001	0.61 (0.51–0.73)	<0.001	0.61 (0.51–0.73)	<0.001
≥5 mm	0.28 (0.25–0.31)	<0.001	0.57 (0.50–0.65)	<0.001	0.56 (0.49–0.64)	<0.001
test for trend *p*	<0.001		<0.001		<0.001	
LNY						
1–14	1		1		1	
15–29	0.74 (0.68–0.81)	<0.001	1.11 (0.98–1.25)	0.089	0.74 (0.66–0.82)	<0.001
≥30	1.02 (0.93–1.11)	0.684	1.81 (1.60–2.03)	<0.001	0.90 (0.81–1.00)	0.061
test for trend *p*	0.011		<0.001		<0.001	
Treatment						
Surgery	1				1	
Surgery + RT	1.68 (1.50–1.88)	<0.001	0.77 (0.67–0.88)	<0.001	0.69 (0.61–0.79)	<0.001
Surgery + CT	3.44 (3.05–3.88)	<0.001	1.35 (1.16–1.57)	<0.001	1.27 (1.09–1.49)	0.002
Surgery + CRT	2.84 (2.62–3.07)	<0.001	0.63 (1.16–1.57)	<0.001	0.55 (0.49–0.62)	<0.001

Abbreviations: PNI: perineural invasion; LVI: lymphovascular invasion; LND: lymph node density; RT: radiotherapy; CT: chemotherapy; CRT: chemoradiotherapy; ECOG Performance Status Scale; NA: not available.

## Data Availability

Restrictions apply to the availability of these data. Data were obtained from the Health and Welfare Data Science Center (HWDC), Ministry of Health and Welfare, Taiwan, and are available from the authors with the permission of HWDC.

## Supplementary Materials

The following supporting information can be downloaded at: https://www.mdpi.com/article/10.3390/cancers17162704/s1, Table S1: Univariate and Multivariate Cox Regression Analyses of Lymph Node Density (LND) and Log Odds of Positive Lymph Nodes (LODDS) for Predicting Overall Survival in Node-Positive OSCC Patients.

## Abbreviations

The following abbreviations are used in this manuscript:

**Abbreviation**	**Full Term**
OSCC	Oral Squamous Cell Carcinoma
OS	Overall Survival
DSS	Disease-Specific Survival
LND	Lymph Node Density
LODDS	Log Odds of Positive Lymph Nodes
AJCC	American Joint Committee on Cancer
BMI	Body Mass Index
ECOG	Eastern Cooperative Oncology Group
PNI	Perineural Invasion
LVI	Lymphovascular Invasion
DOI	Depth of Invasion
ENE	Extranodal Extension
LNY	Lymph Node Yield
RT	Radiotherapy
CT	Chemotherapy
CRT	Chemoradiotherapy
KM	Kaplan–Meier
HR	Hazard Ratio
CI	Confidence Interval
IQR	Interquartile Range
SD	Standard Deviation
TCRD	Taiwan Cancer Registry Database
IRB	Institutional Review Board
ICD-O-3	*International Classification of Diseases for Oncology*, Third Edition
HWDC	Health and Welfare Data Science Center
