# Supplementary material for: The Cervical Lymph Node Positive Metastatic Probability Is a Significant Predictor of Survival for Oral Squamous Cell Carcinoma—A Nationwide Study
Source: Cancers (Basel). 2025 Aug 20;17(16):2704. doi: 10.3390/cancers17162704 (PMC12385004; doi:10.3390/cancers17162704)
Supplement: Supplementary file 1 [file cancers-17-02704-s001.zip › cancers-3753786-supplementary.pdf]

Supplemental Table S1. Univariate and Multivariate Cox Regression Analyses of Lymph Node Density (LND) and Log Odds of Positive Lymph Nodes (LODDS) for Predicting Overall Survival in Node-Positive OSCC Patients.

	Univariate Model		Multivariable Model 1		Multivariable Model 2	
Variables	HR (95% CI)	P value	HR (95% CI)	P value	HR (95% CI)	P value
LND						
<=0.05	1.00		1.00			
＞0.05	1.89 (1.60-2.17)	<0.001	1.74 (1.56-1.93)	<0.001		
LODDS						
< -4	1.00		1.00		1.00	
-4 to -3.5	0.77 (0.49-1.20)	0.247			1.00 (0.62-1.63)	0.993
-3.5 to -2.5	0.88 (0.59-1.32)	0.542			1.08 (0.70-1.67)	0.735
> -2.5	1.51 (1.01-2.24)	0.042			1.96 (1.26-3.05)	0.003
**test for trend p**	<0.001				<0.001	

Multivariable models adjusted for demographic (age, sex, BMI), clinical (ECOG PS), pathological (pT stage, tumor subsite, histological grade, PNI, LVI, margin status), and treatment-related variables (LNY and treatment modality). Model 1 focused on LND, while Model 2 focused on LODDS.
